# Older Patients’ Enthusiasm to Use Electronic Mail to Communicate With Their Physicians: Cross-Sectional Survey

**DOI:** 10.2196/jmir.1143

**Published:** 2009-06-16

**Authors:** Hardeep Singh, Sarah A Fox, Nancy J Petersen, Anila Shethia, Richard L Street

**Affiliations:** ^4^Department of CommunicationTexas A&M UniversityCollege StationTexasUSA; ^3^Center for Community Partnerships in Health Promotion Division of General Internal Medicine and Health Services ResearchDavid Geffen School of MedicineUniversity of California at Los AngelesLos AngelesCAUSA; ^2^Center of Inquiry to Improve Outpatient Safety through Effective Electronic Communication at the Michael E DeBakey Veterans Affairs Medical CenterHoustonTexasUSA; ^1^Houston VA Health Services Research and Development (HSR&D) Center of ExcellenceMichael E DeBakey Veterans Affairs Medical Center and the Section of Health Services ResearchDepartment of MedicineBaylor College of MedicineHoustonTexasUSA

**Keywords:** Electronic mail, doctor-patient communication, Internet, doctor-patient relationship

## Abstract

**Background:**

Recent evidence indicates increased access to and use of Internet and non-healthcare-related email by older patients. Because email adoption could potentially reduce some of the disparities faced by this age group, there is a need to understand factors determining older patients’ enthusiasm to use email to communicate with their physicians. Electronic mail (email) represents a means of communication that, coupled with face-to-face communication, could enhance quality of care for older patients.

**Objective:**

Test a model to determine factors associated with older patients’ enthusiasm to use email to communicate with their physicians.

**Methods:**

We conducted a secondary data analysis of survey data collected in 2003 for two large, longitudinal, randomized controlled trials. Logistic-regression models were used to model the dichotomous outcome of patient enthusiasm for using email to communicate with their physicians. Explanatory variables included demographic characteristics, health status, use of email with people other than their physician, characteristics of the physician-patient relationship, and physician enthusiasm to use email with patients.

**Results:**

Participants included a pooled sample of 4059 patients over 65 years of age and their respective physicians (n = 181) from community-based practices in Southern California. Although only 52 (1.3%) patient respondents reported that they communicated with their physician by email, about half (49.3%) expressed enthusiasm about the possibility of using it. Odds of being enthusiastic decreased with increased age (by 0.97 for each year over 66) but were significantly higher in African Americans (OR = 2.1, CI = 1.42 - 3.06), Hispanics (OR = 1.6, CI = 1.26 - 2.14) and men (OR = 1.3, CI = 1.1 - 1.5).

A perception of better communication skills of their physician, lower quality of interaction with physician in traditional face-to-face encounters, and physician enthusiasm to use email with patients were significantly associated with an enthusiasm to use email. Patients who did not use email at all were less enthusiastic compared to those who used email for other reasons. Half of the physician respondents were not enthusiastic about communicating with patients using email.

**Conclusions:**

Despite perceived barriers such as limited access to the Internet, older patients seem to want to use email to communicate with their physicians.

## Introduction

Good communication between patients and physicians is a cornerstone of modern, high quality health care. Recent advances in communication technology are generating a variety of communication exchanges that could complement or replace more traditional face-to-face visits and telephone calls.

Because of its pervasiveness and relative ease of use, electronic mail (email) offers a potentially valuable resource for augmenting and improving communication between physicians and patients [1]. Even so, email communication remains an untapped resource in health care [2]. Although many physicians believe email communication can enhance chronic-disease management and improve continuity of care [3], its adoption is generally low [4-6]. Factors such as lack of reimbursement, fears about negative impact on their own quality of life, and concerns surrounding the risk of liability[7,8], reduce physician enthusiasm to use email. Conversely, patient enthusiasm to use email appears to be high [9], even though their actual use of email to communicate with physicians is generally low [4,10]. Given that patient enthusiasm to use email represents the motivational catalyst that could lead to its more routine use, this investigation examined factors affecting enthusiasm among elderly patients to communicate with physicians using email. This age group is at risk of poor communication with physicians, in spite of having multiple co-morbidities, and is slower to adopt new communication technologies.

Despite effective doctor-patient communication being paramount for patients over 65 years of age [11,12], we are not aware of any studies of email use (or enthusiasm to use email) in health care that have specifically studied this age-group. Although activities such as Internet use and email are generally more prevalent in younger age groups [13,14], older adults may also appreciate having this additional medium to communicate their concerns [15].

While older patients may have more barriers that limit their use of the Internet, there exist several reasons why they could be enthusiastic about using email with their physicians. For example, traditional face-to-face communication encounters between older patients and their physicians may be ineffective if the discussions do not raise all issues of concern. Moreover, physicians are often less responsive to the psychosocial issues raised during visits by older patients than to similar concerns of younger patients [16]. Subsequent follow-up email correspondence could also allow older patients to raise additional topics of concern or identify unmet psychosocial needs. Finally, older patients face several communication challenges due to their capacity to remember and follow complex instructions and, thus, a follow-up email summarizing the visit can reinforce instructions [12].

Recent evidence indicates increased access to and use of Internet and non-healthcare-related email by older patients [17]. Because email adoption could potentially reduce some of the disparities faced by this age-group, there is a need for understanding factors determining their enthusiasm to use email with their physicians. In addition, a high level of patient enthusiasm, accompanied by the rapid diffusion of technology in this age group, could also be used as grounds for reimbursement-related policy changes.

We hypothesized that, in addition to demographics and familiarity with technology, older patients’ enthusiasm to use email to communicate with their physicians would depend on their health needs and the quality of their relationships. Specifically, patients with greater medical needs and a stronger relationship with their physicians will be more enthusiastic about using email as a communication tool. Our main study objective was to test a model to determine factors associated with older patients’ enthusiasm to use email with their physicians. Secondarily, we examined factors associated with physicians’ enthusiasm to communicate with their patients using email.

## Methods

We conducted a secondary data analysis of survey data collected for two large randomized controlled trials in Southern California, known as Communication in Medical Care 2 and 3 (CMC 2 and 3), which were designed to study and improve physician-patient communication regarding cancer screening. (See Fox et al [18] for background study, CMC 1.)

CMC 2 was a community-based, longitudinal, randomized controlled trial conducted between 1998 and 2003 that involved 111 primary care physicians practicing full time in community-based office practices in Los Angeles County. Patients were recruited from these physicians’ practices. The patients were non-institutionalized and between 50 and 80 years of age; were physically and mentally capable of completing a 30-minute interview; and did not have a history of breast, cervical, colorectal, or prostate cancer. Only patients aged 65 - 80 were included in this analysis. Baseline and exit data were collected in 2000 and 2003 through 20-minute telephone interviews with physicians and 30-minute telephone interviews with patients. Data were collected on the patients’ health care access and utilization; general demographics; mental and physical health; patterns of physician-patient communication, including use of, and enthusiasm for, using email; and certain characteristics of patient-physician relationships. Survey-response rate for participants, after being enrolled, was 72%.

CMC 3 was focused on patients aged 65 - 79. Their 80 primary care physicians practiced in community-based practices in Southern California (excluding Los Angeles County). Baseline and exit data were collected in 2003 and 2006 through 20-minute telephone interviews with physicians and 30-minute telephone interviews with patients. A total of 5978 patients participated in both the original studies. Overall, the CMC 2 sample of patients from Los Angeles County represented a range of socioeconomic levels and was more diverse in its ethnic representation, whereas the CMC 3 sample represented more suburban areas, was predominantly white, and had somewhat higher socioeconomic levels. Over 11,000 people were contacted for recruitment over the telephone in 2003, from whom we obtained 3188 completed interviews for analysis.

### Data Analysis

To allow cross-sectional analyses for our main study objective of determining the factors associated with older patients’ enthusiasm to use email with their physicians, we pooled data from CMC 2 exit surveys and the CMC 3 baseline survey in 2003.

The study population of patients was limited to those over 65 years of age in 2003. For patients from the CMC 2 survey, age was determined by adding 3 years to the patient’s age in the CMC 2 baseline survey conducted in 2000. For patients from the CMC 3 survey, we used their age at the time of the CMC 3 baseline survey. The proportions of patients and physicians who used or were enthusiastic about using email as a communication tool were calculated from the pooled 2003 data.

**Figure 1:**
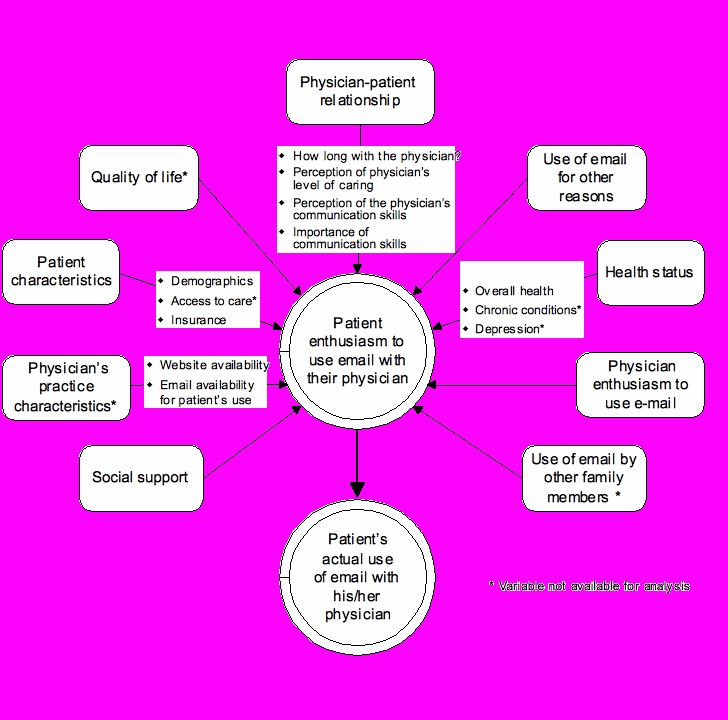
Potential determinants of older patients’ enthusiasm to use email communication with their physicians

**Figure 2:**
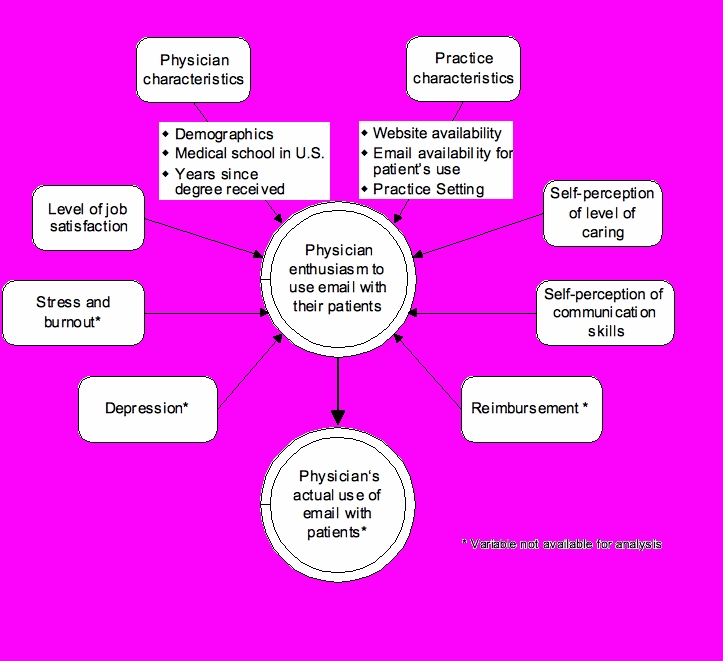
Potential determinants of physician enthusiasm to use email communication with their patients

Figure 1 illustrates the potential factors we considered to derive the explanatory variables explaining patient enthusiasm in our model. These included demographic variables (patient age, race, gender, and marital status), health status, social support, quality of life, access to care, use of general email (such as with people other than their physicians), characteristics of physician-patient relationship, and physician’s enthusiasm to use email. Because physician enthusiasm could depend on additional factors, we used explanatory variables, including the clinician’s age, race, gender, time in the United States, level of job satisfaction, practice characteristics, self-perceptions with respect to caring for their patients, and self-perceptions of communication skills (Figure 2). For the model of physician enthusiasm, several variables were excluded because of their high correlation with other variables in the model. For example, the number of years since the physician had received his or her medical degree was highly correlated with physician age and was therefore excluded from the model. Both Figure 1 and Figure 2 show additional variables we considered but excluded because one or both surveys did not collect any information about them.

We first used univariate analysis to identify potential explanatory variables of enthusiasm for both patients and physicians using variables collected in both surveys. Chi-square analysis was used to compare the categorical variables, and the *t* test was used for continuous variables. Separate logistic-regression models were used to model the dichotomous outcome as to whether

there was enthusiasm to use email by patients and physicians. For the cross-sectional analysis of patient enthusiasm, we conducted the logistic regression using generalized estimating equations (GEE) methodology. To account for potential correlations among patients with the same physician, patients were nested within their own physician.

## Results

For the cross-sectional analyses, we studied survey responses of 4059 patients over 65 years of age to evaluate the determinants of their enthusiasm to use email with their health care providers. Table 1 shows characteristics of the study population of patients in the pooled sample. The mean age was 73.1 (SD 4.1). Non-Hispanic whites represented 81.1% of the study population, with Hispanics representing 11.9%, African Americans representing 3.8%, and other races representing 3.2%. Almost all had insurance coverage through Medicare, Medi-Cal, government or military insurance, or private insurance. Three-quarters (75.9%) considered themselves in good, very good, or excellent health. On average, the participants had been patients of their current physicians for 7.8 years (SD 6.4). Although most patients felt their physician was always respectful of them (91.2%), only 62.0% thought their physician always allowed enough time to talk. Most patients (89%) rated their provider as having very good, excellent, or “better than most” communication skills.

**Table 1:** Characteristics of patients over age 65 in the pooled sample from the 2003 CMC 2 exit questionnaire and 2003 CMC 3 baseline questionnaire

Characteristic				Number of patients (Mean)	% (Standard deviation)
**Demographics**					
	**Average Age** in years (n = 4059)			(73.1)	(4.1)
	**Age** (n = 4059)				
		66 - 69		949	23.4
		70 - 74		1557	38.4
		75 - 79		1366	33.7
		80 and older		187	4.6
	**Gender** (n = 4059)				
		Male		1671	41.2
		Female		2388	58.8
	**Race** (n = 4033)				
		Non-Hispanic white		3271	81.1
		African American		155	3.8
		Asian/Other		128	3.2
		Hispanic		479	11.9
	**Marital status** (n = 4052)				
		Married or living as married		2496	61.6
		Not married		1556	38.4
**Insurance**^a^ (n = 4059)					
		Has medical insurance		3985	98.2
**Health Status**					
	Patient’s rating of his/her own health (n = 4050)				
		Fair, poor		974	24.1
		Good		1301	32.1
		Very good		1321	32.6
		Excellent		454	11.2
**Use of Email for Other Reasons**					
	Patient (n = 4059)			1456	35.9
**Physician/Patient Relationship**					
	**Average years as clinician’s patient** (n = 4050)			(7.8)	(6.4)
	**Perception of physician’s level of caring:**				
		Patient thinks physician is respectful (n = 3458)			
			Never, sometimes	82	2.4
			Usually	223	6.5
			Always	3153	91.2
		Patient thinks physician allows enough time to talk (n = 4047)			
			Never, sometimes	466	11.5
			Usually	1074	26.5
			Always	2507	62
			
	**Perception of physician’s communication skills:**				
		Importance of good communication skills of primary care provider (n = 4033)			
			Somewhat important	126	3.1
			Very important	1730	42.9
			Extremely important	1384	34.3
			More important than anything else	793	19.7
		Patient’s rating of provider’s communication skills (n = 4047)			
			Fair, good	447	11.1
			Very good	1003	24.8
			Excellent	1600	39.5
			Better than most	997	24.6

^a^A patient was defined as having insurance if he or she indicated that they had Medi-Cal, Medicare, government or military insurance, or private insurance.

Few patients (1.3%) indicated that they communicated with their physician through email. Of patients who did not use email to communicate with their physicians, half (49.3%) reported they were enthusiastic about doing so. Table 2 shows the relationship between the potential predictors and the patient’s enthusiasm to use email in a GEE logistic regression model of the pooled population. For each year of increase in patient age, the odds of being enthusiastic decreased by 0.97. African Americans and Hispanics were 2.1 times and 1.6 times more enthusiastic than non-Hispanic whites, respectively. Men had odds that were 1.25 times higher than those of women. Patients who did not use email in general had lower odds (0.17) of being enthusiastic than those who did. Other patient characteristics, such as the patient’s marital status and rating of health status were not significant. The CMC 2 sample was more likely to be enthusiastic about using email, probably because they were younger than those in the CMC 3 sample.

**Table 2:** Logistic regression analysis of patient enthusiasm to use email in 2003 (all patient characteristics were significant in univariate analysis)

Patient Characteristics		Odds Ratio	95% Confidence Interval	*P* Value
**Age**		0.97	0.95 - 0.99	< .001
**Race** (reference group is non-Hispanic white)				
	African American	2.08	1.42 - 3.06	< .001
	Hispanic	1.64	1.26 - 2.14	< .001
	Asian	1.35	.87 - 2.09	.18
**Gender** (reference group is female)				
	Male	1.25	1.06 - 1.47	.01
**Marital status** (reference group is not married)				
	Married	1.08	0.89 - 1.31	.45
**Rating of their health status** (reference group is excellent)				
	Fair or poor	0.83	0.63 - 1.10	.19
	Good	0.95	0.74 - 1.24	.72
	Very good	0.99	0.76 - 1.30	.95
**Years as a patient of their physician**		1.00	1.00 - 1.02	.11
**Use of email for other reasons** (reference group is “do use email for other purposes”)				
	Do not use email for other purposes	0.17	0.15 - 0.20	<.001
**Rating of the importance of physician’s communication skills** (reference group is most important)				
	Somewhat important	0.84	0.51 - 1.38	.49
	Very important	0.92	0.77 - 1.12	.41
	Extremely important	1.01	0.82 - 1.24	.91
**Rating of their physician’s communication skills** (reference group is fair or good)				
	Better than most	1.58	1.17 - 2.14	.01
	Excellent	1.36	1.05 - 1.76	.02
	Very good	1.20	0.92 - 1.57	.18
**Rating of whether physician allows enough time to talk** (reference group is always)				
	Never	1.14	0.85 - 1.51	.39
	Usually	1.43	1.20 - 1.72	<.001
**Enthusiastic about communicating using email** (reference group is physician is not enthusiastic about communicating using email)		1.31	1.11 - 1.54	.001
**Survey group** (reference group is CMC 3 baseline survey)				
	CMC 2 exit survey	1.27	1.03 - 1.57	.03

The regression model found several physician and patient-physician relationship characteristics to be significant. First, patients whose physician was enthusiastic about using email were 1.3 times more likely to be enthusiastic than patients whose physician was not enthusiastic. Second, patients who rated their physician’s communication skills high (better than most) were 1.58 times more likely to be enthusiastic compared to those who rated their physician’s communication skills fair/good. Finally, patients whose physicians *usually* allowed enough time to talk were 1.4 times *more* likely to be enthusiastic than patients whose physician *always* allowed enough time to talk. Factors such as duration of the patient-physician relationship did not correlate highly with enthusiasm.

Regarding physicians’ enthusiasm to use email (Table 3), approximately half (51.7%) responded that they were not at all enthusiastic about communicating with patients using email. Just over a quarter (26.7%) were somewhat enthusiastic, while only 10% were very or extremely interested in email communication.

**Table 3:** Characteristics of 181 physicians in the pooled sample from the 2003 CMC 2 exit and 2003 CMC 3 baseline surveys

Physician Characteristic (n = 181)		n (Mean)	% (Standard deviation)
**Average age**		(49.4)	(9.0)
**Average years in US**		(38.8)	(15.9)
**Avg. years in current practice setting**		(12.4)	(8.5)
**Age**			
	< 40	51	28.2
	40 - 49	48	26.5
	50 - 54	38	21.0
	55 and older	44	24.3
**Gender**			
	Male	136	75.1
	Female	45	24.9
**Race**			
	Non-Hispanic white	93	51.4
	African American, other	12	6.6
	Asian/Pacific Islander	40	22.1
	Hispanic	36	19.9
**Area of birth**			
	United States	100	55.3
	Mexico, Central America, South America	19	10.5
	Asia, India	35	19.3
	Other	27	14.9
**Practice setting**			
	Private solo practice	97	53.6
	Private group practice	49	27.1
	HMO, other	35	19.3
**Specialty**			
	Family practice/general practice	96	53.0
	Internal medicine	85	47.0
**Use of email for other reasons**			
	Physician	137	75.7

Table 4 shows factors associated with physician enthusiasm to use email according to the logistic regression analysis of the pooled CMC 2 exit and CMC 3 baseline datasets. Notably, the odds of a physician being enthusiastic were 4.96 times higher for physicians who were somewhat or very dissatisfied with their current work setting compared to physicians who were very satisfied. Physicians who reported that they *always* provided educational materials to patients were significantly less enthusiastic about using email than physicians who reported that they *usually* provided those materials (OR = 0.28). There was no association between physician enthusiasm and demographic characteristics, such as the physician’s age and gender nor practice characteristics, such as setting or years in current practice. There was also no significant association between physician enthusiasm and the rating of their communication skills or the likelihood that they would build a partnership with their patients.

**Table 4:** Logistic regression analysis of physician enthusiasm to use email in 2003 (all physician characteristics were significant in univariate analysis)

Physician Characteristics		Odds Ratio	95% Confidence Interval	*P* Value
**Age** (reference group is under 40)				
	40 - 49	0.92	0.31 - 2.73	.88
	50 - 54	1.05	0.32 - 3.49	.94
	55 and older	0.84	0.24 - 2.98	.79
**Race** (reference groups is non-Hispanic white)				
	African American	1.18	0.23 - 6.15	.85
	Hispanic	2.46	0.84 - 7.17	.10
	Asian	2.29	0.80 - 6.52	.12
**Gender** (reference group is male)				
	Female	0.37	0.14 - 1.00	.05
**Years in US** (reference group is less than 25 years)				
	25 or more years	1.31	0.47 - 3.63	.60
**Years in current practice setting** (reference group is less than 5 years)				
	5 - 9	1.87	0.55 - 6.41	.32
	10 - 19	2.39	0.78 - 7.29	.13
	20 or more	0.94	0.26 - 3.38	.92
**Current practice setting** (reference group is private solo practice)				
	Private group practice	1.62	0.65 - 4.05	.31
	HMO or other	0.78	0.25 - 2.43	.66
**Specialty** (reference group is internal medicine)				
	Family practice	1.79	0.57 - 5.68	.32
	General internal medicine	2.11	0.47 - 9.54	.33
**Use of email for other reasons** (reference group is “do use email for other purposes”)				
	Do not use email for other purposes	0.62	0.26 - 1.50	.29
**Rating of their communication skills with older patients** (reference group is very good)				
	Better than most	1.09	0.39 - 3.03	.87
	Excellent	0.71	0.28 - 1.81	.47
	Fair or good	1.52	0.42 - 5.46	.52
**Rating of the importance of their communication skills** (reference group is somewhat or very important)				
	Most important	1.15	0.54 - 2.43	.73
**Rating of their satisfaction with their current work setting** (reference group is very satisfied)				
	Somewhat or very dissatisfied	4.96	1.48 - 16.68	.01
	Somewhat satisfied	2.21	0.98 - 5.01	.06
**Provides educational materials** (reference group is usually)				
	Always	0.28	0.08 - 0.99	.05
	Never or sometimes	0.79	0.33 - 1.90	.60
**Builds partnership with patients** (reference group is usually)				
	Always	0.83	0.37 - 1.88	.66
	Never or sometimes	0.45	0.10 - 2.04	.30
**Provides ample time to talk** (reference group is usually)				
	Always	2.22	0.93 - 5.28	.07
	Never or sometimes	0.35	0.10 - 1.19	.09
**Survey group** (reference group is CMC3 baseline survey)				
	CMC 2 exit survey	1.43	0.49 - 4.21	.51

## Discussion

Electronic communication holds the potential to enhance the patient-physician relationship and quality of care by expanding the opportunities for patients and physicians to interact [8,19,20]. Older patients would likely benefit most from electronic correspondence with their physicians. We found that nearly half the patients surveyed were indeed enthusiastic about using email with physicians. Enthusiasm to use email was affected by several factors that may have significant implications for future research, clinical practice, and policy decisions.

First, even though overall use of email with health care providers was low, older patients and especially non-whites were likely to adopt this technology if given the opportunity. Our findings strongly suggest consideration of email as a medium to overcome communication barriers affecting this population. Public interest in and demand for expanding the use of this technology in the senior population [17] could have significant implications for reimbursement policies. Some insurance carriers reimburse physicians for certain types of email, and the American College of Physicians advises Medicare to reimburse selected use of email [21]. Enthusiasm for email use is likely to grow with increasing access to the Internet and might provide a basis for future reimbursement-related policy changes for the Medicare population.

Second, our study suggests that the patient-physician relationship is relevant in determining patient enthusiasm to use email with a physician. Our study supports findings from a recent study which found that certain aspects of the patient-provider relationship affected interest in the use of computerized patient portals [22]. Consistent with previous research, increasing age and less familiarity with technology were negatively related to enthusiasm [17]. Although we found that enthusiasm to use email among older adults decreased with increasing age, it still remained relatively high overall.

Third, we noted two unexpected findings related to demographics. First, subjects with self-reported poor health status were not highly enthusiastic about using email, contrary to findings reported in previous literature [13,14]. Second, we found that non-white patients were more enthusiastic than white patients about using email, also in contrast to previous findings [13]. Because non-whites generally receive less positive talk (positive talk includes more verbal behavior, agreements, encouragement, and reassurance) and information even within the same medical practice [23], their use of email may overcome some of the communication barriers they face. Being a less socially intimidating forum, an electronic medium could bolster the quality of patient-physician communication, since it might encourage older adults to ask questions and provide vital information more readily than during face-to-face communication [14]. This may be especially relevant in older men; men in general ask fewer questions, receive less positive talk, and are less likely to be included in discussion than women [23]. These reasons may explain why older men are more enthusiastic about using an alternative medium such as email to communicate with physicians.

Adoption by older patients of email as a tool to communicate with their physicians might also depend on the attitudes and beliefs of physicians and the value they place on communicating electronically. Previous work shows the criteria applied by physicians to use email remain subjective and depend on factors besides patient barriers (eg, a patient’s access to the Internet), such as reimbursement for time spent writing email [3,24,25]. Although physician characteristics, such as demographic [5] and time and place of training, and practice characteristics, such as the setting and availability of a practice website, were expected to affect enthusiasm for email use, our findings did not substantiate this expectation. The quality of patient-physician communication may also be affected by a physician’s morale and job stress [26]. Physicians dissatisfied with their careers cite problems in relationships with their patients and difficulties in caring for them, in addition to problems in communicating with specialists [27]. We expected these physicians to have less enthusiasm for using email but found quite the opposite. A partial explanation for this could be that these physicians found the prospects of an alternative medium of communication with patients especially valuable in addressing problem areas of communication within their practices. Furthermore, both confidentiality issues, such as those posed by HIPAA,and reimbursement-related issues pose additional barriers which dampen physician enthusiasm [7,8]. For example, physicians may have concerns that email will be too time consuming and not worth their time if they are not compensated [28].

Our findings also have implications for strategies to improve the use of email by older patients and their physicians. Availability of the Internet through community resources and efforts to engage family members in the process could significantly affect the use of email by older patients whose access to technology may be limited. Physician enthusiasm could be increased by having continuing medical education programs on electronic communication with a focus on specific barriers noted by physicians (eg, HIPAA limits).

Our study has certain limitations. Our analysis was based on a cross-sectional secondary look at existing data, and data on certain factors that could have played a role in determining enthusiasm (eg, use of email by other family members, reimbursement to physicians) were not collected at the outset. Secondly, while patient enthusiasm may be higher now than it was in 2003, factors determining patient enthusiasm are likely not to have changed dramatically. Our strengths include a large sample size drawn from a large, populous area; a diverse population that is representative of the region; and the inclusion of both genders. We also have a wide representation of primary care with diverse sets of physicians.

In conclusion, our study lends support to our hypothesis that, in addition to factors related to patient demographics and familiarity with technology, enthusiasm to use email depends upon the quality of existing relationships between patients and physicians. We found that older patients, especially non-whites, are highly likely to adopt this technology, but that factors arising from their interactions with physicians in traditional face-to-face encounters or their physician’s interest in the use of email could adversely affect their interest. Significant opportunities exist to use electronic tools to overcome some communication barriers affecting older patients. Further study on whether the adoption of email can reduce communication-related health disparities in the older non-white population is warranted. Public interest and demand in expanding the use of email could potentially lead to changes in reimbursement policies concerning the use of email.

## Abbreviations

CMC: communication in medical care
GEE: generalized estimating equations
HIPAA: Health Insurance Portability and Accountability Act
